# Prevalence and factors associated with problematic use of smartphone in high school students from southern Brazil

**DOI:** 10.1590/0102-311XEN140024

**Published:** 2025-07-25

**Authors:** Bruno Pedrini de Almeida, Samuel C. Dumith, Michael Pereira da Silva

**Affiliations:** 1 Programa de Pós-graduação em Ciências da Saúde, Universidade Federal do Rio Grande, Rio Grande, Brasil. Programa de Pós-graduação em Ciências da Saúde Universidade Federal do Rio Grande Rio Grande Brazil

**Keywords:** Smartphone, Adolescent, Risk Factors, Sleep Quality, Smartphone, Adolescente, Fatores de Risco, Qualidade do Sono, Teléfono Inteligente, Adolescente, Factores de Riesgo, Calidad del Sueño

## Abstract

This study investigated the prevalence and factors associated with problematic smartphone use (PSU) in southern Brazil. A total of 411 high school students at a federal institute participated in this research. Smartphone addiction was highlighted as the dependent variable and was assessed using the *Smartphone Addiction Scale-Short Version*, classifying students of both sexes who scored ≥ 33 on this scale as having PSU. Information on gender, skin color, socioeconomic status, level of physical activity, participation in physical education classes, screen time, and history of binge drinking were organized as independent variables. Poisson regression was used to verify the factors associated with PSU, showing prevalence ratios (PR) with a 95% confidence interval (95%CI). The prevalence of PSU was 34.3% (40.7% female). Adjusted analysis showed significant associations with risk factors (female sex PR = 1.40; 95%CI: 1.06-1.85; screen time PR = 1.48; 95%CI: 1.20-1.82; and history of binge drinking PR = 1.35; 95%CI: 1.02-1.79) and protective factors (higher socioeconomic status PR = 0.88; 95%CI: 0.77-0.99; longer participation in physical education classes PR = 0.73; 95%CI: 0.54-0.99) for PSU. Moreover, students with PSU had worse sleep quality (PR = 1.17; 95%CI: 1.02-1.34), and this effect was more significant in the physically inactive ones (PR = 1.50; 95%CI: 1.13-1.98). Identifying the factors associated with PSU can help raise awareness of the repercussions of this behavior.

## Resumo

Este estudo analisou a prevalência e os fatores associados ao uso problemático de *smartphones* (UPS) no sul do Brasil em 411 estudantes do Ensino Médio de um instituto federal. O UPS serviu como variável dependente, avaliada por meio da *Smartphone Addiction Scale-Short Version* [Escala de Dependência de Smartphone-Versão Curta]. Estudantes de ambos os sexos que pontuaram ≥ 33 nessa escala foram classificados como fazendo UPS. Informações sobre sexo, cor da pele, nível socioeconômico, atividade física, participação em aulas de educação física, tempo de tela e histórico de consumo excessivo de álcool foram organizadas como variáveis independentes. Uma regressão de Poisson (com razões de prevalência [RP] com intervalo de 95% de confiança [IC95%]) serviu para verificar os fatores associados ao UPS. A prevalência de UPS foi de 34,3% (40,7% do sexo feminino). A análise ajustada mostrou associações significativas com fatores de risco (sexo feminino: RP = 1,40; IC95%: 1,06-1,85; tempo de tela: RP = 1,48; IC95%: 1,20-1,82; e história de consumo excessivo de álcool: RP = 1,35; IC95%: 1,02-1,79) e fatores de proteção (maior nível socioeconômico: RP = 0,88; IC95%: 0,77-0,99; maior participação nas aulas de educação física: RP = 0,73; IC95%: 0,54-0,99) para UPS. Os alunos com UPS apresentaram pior qualidade do sono (RP = 1,17; IC95%: 1,02-1,34), sendo que esse efeito foi mais significativo entre pessoas fisicamente inativas (RP = 1,50; IC95%: 1,13-1,98). Identificar os fatores associados ao UPS pode ajudar a aumentar a conscientização sobre as repercussões desse comportamento.

## Resumen

Este estudio analizó la prevalencia y los factores asociados al uso problemático de teléfonos inteligentes (UPTI) en el sur de Brasil. En esta investigación, participaron 411 estudiantes de nivel medio de un instituto federal. Se destacó la UPTI como variable dependiente, la cual se evaluó mediante la Smartphone Addiction Scale-Short Version *[Escala de Adicción al Teléfono Inteligente-Versión Corta]*. Los estudiantes de ambos sexos que obtuvieron una puntuación de ≥ 33 en esta escala se clasificaron como con un UPTI. Las informaciones sobre el sexo, el color de la piel, el nivel socioeconómico, el nivel de actividad física, la participación en clases de educación física, el tiempo frente a la pantalla y los antecedentes de consumo excesivo de alcohol se organizaron como variables independientes. Se utilizó la regresión de Poisson para verificar los factores asociados al UPTI, presentando razones de prevalencia (RP) con un intervalo de 95% de confianza (IC95%). La prevalencia de UPTI fue del 34,3% (40,7% entre mujeres). El análisis ajustado mostró asociaciones significativas con los factores de riesgo (sexo femenino: RP = 1,40; IC95%: 1,06-1,85; tiempo de pantalla: RP = 1,48; IC95%: 1,20-1,82; antecedentes de consumo excesivo de alcohol: RP = 1,35; IC95%: 1,02-1,79) y factores protectores (nivel socioeconómico más alto: RP = 0,88; IC95%: 0,77-0,99; mayor participación en las clases de educación física: RP = 0,73; IC95%: 0,54-0,99) para el UPTI. Además, los estudiantes con UPTI tenían una peor calidad de sueño (RP = 1,17; IC95%: 1,02-1,34); este efecto fue más significativo entre aquellos que eran físicamente inactivos (RP = 1,50; IC95%: 1,13-1,98). Identificar los factores asociados con el UPTI puede ayudar a crear conciencia sobre las repercusiones de este comportamiento.

## Introduction

Smartphones are increasingly integrated into people’s daily lives, and they are the most consumed electronic devices in developed and developing countries ^1^. The technological advances of this type of device enable users to spend much of their time on leisure activities and services, searching for information of interest in various internet content, interacting in electronic games, and chatting with friends ^2^.

In 2021, 90% of Brazilian households had a smartphone, which is expected to increase by 13 million by 2028 ^3^^,^^4^. Users seek autonomy, identity, and prestige via smartphone interaction, especially during adolescence, in which they are more susceptible to make decisions without weighing up the consequences due to relatively lower self-control ^5^^,^^6^. This characteristic can be manifested when adolescents come into contact with the varied entertainment possibilities available by using the device.

In this context, problematic smartphone use (PSU) is a phenomenon related to the maladaptive use of the device ^7^. This behavior becomes specific because it goes beyond a simple reflection of the total time spent interacting with the smartphone. It recognizes that aspects such as tolerance, avoidance of problems, withdrawal, desire, and social motivation are part of the PSU construct, meeting a pattern of dependence that is related to impulsive disorders and can negatively influence users’ lives ^8^^,^^9^.

It is estimated that 23.3% of children and adolescents worldwide have PSU ^10^, and in Brazil there is a higher magnitude with prevalences ranging from 53% to 70%, although different scales have been used to measure behavior ^11^^,^^12^^,^^13^. Although not conclusive, emerging evidence indicates that females, older adolescents, length of exposure, and the pattern of use involving social interactions over the internet can be considered risk factors ^10^. Moreover, PSU has been indicated as an essential risk factor for mental and behavioral health outcomes. In this sense, sleep quality is the target of research investigating the impact of exposure to smartphones.

More than 70% of adolescents do not reach the daily recommendation for ideal sleep time ^14^. Especially during the school term, their possession of the device is associated with sleep-related problems, which suggests that a more dependent relationship would imply significant negative consequences ^15^.

Given the proportionality of smartphone use in Brazil and the possible effects of this technology on users’ daily lives, this article aimed to verify the prevalence and factors associated with PSU and its association with sleep quality in a sample of students from southern Brazil.

## Methods

### Study design

This is a cross-sectional study of the *Health at School* project and was approved by the Research Ethics Committee of the Federal University of Rio Grande (protocol n. 26359019.0.0000.5324). The project surveyed students from the 1st to the 3rd grade of high school at the Federal Institute of Education, Science and Technology of Rio Grande do Sul (IFRS, acronym in Portuguese) in southern Brazil ^16^. The questionnaires were administered in October 2022 by a trained team using tablet computers and stored in the REDCap Mobile App (https://projectredcap.org/software/mobile-app/).

### Participants

Students of both sexes, aged 15 to 22, who were regularly attending high school were included, and those with cognitive limitations that prevented them from completing the self-administered questionnaires were excluded. From 502 invited students, the sample comprised 411, resulting in an 81.8% overall survey response rate.

### Measures

The structured questionnaire included sociodemographic (gender, age, skin color, socioeconomic status) and behavioral factors (screen time, physical activity, time spent in physical education classes, and binge drinking), patterns of smartphone use, PSU, and sleep quality.

#### Sociodemographic factors

Skin color was divided into white, black, yellow, and mixed-race and grouped into white/yellow and mixed-race/black for later analysis. Socioeconomic status was evaluated by the number of consumer goods participants had in their houses. A “goods index” was created based on items that had a coefficient greater than 0.20 in the covariance matrix, which the highest coefficients were, respectively: number of air conditioners (0.52), number of bathrooms in the house (0.47) and number of cars (0.44). This variable was operationalized in quartiles (quartile 1 = lowest; quartile 4 = highest).

#### Behavioral factors

Screen time was assessed by the question, “How many hours per day do you usually do activities, other than watching TV, such as using a computer, playing video games, or doing other activities while sitting down? (Not counting Saturdays, Sundays, holidays, or time spent sitting at school)” ^17^. The variable was organized into up to 2 hours per day, between 2 and 4 hours per day, and > 4 hours per day.

Physical activity level (PAL) comprised out-of-school physical activity practice over the last week ^18^. Participants were classified as inactive (no physical activity), insufficiently active (from 1 to 299 minutes per week), and active (≥ 300 minutes per week).

The student’s participation in physical education classes was assessed using the following alternatives: does not participate; participates less than 30 minutes/class; participates between 30 and 59 minutes/class; and participates more than 60 minutes/class. For analysis purposes, this variable was grouped into < 30 minutes/class and ≥ 30 minutes/class.

Binge drinking was defined as consuming ≥ 5 doses for males or ≥ 4 doses for females of alcohol at least one day over the past 30 days ^19^.

#### Pattern of smartphone use and PSU

The pattern of smartphone use was assessed using general questions, such as: “Do you use a smartphone?” and “Do you have your own smartphone?”; “How long have you been using the device?”; “How much time do you use it each day?”; “For what purpose do you use your smartphone the most (social networks, games, study, work, music content, other)?”; “Do you usually use your smartphone in bed?” and, “Do you think smartphone use can hinder your life at some point?”.

The *Smartphone Addiction Scale-Short Version* (SAS-SV) assessed the PSU (α = 0.81) ^11^^,^^20^. In this 10-item scale, each item scores from 1 to 6 points, with answers related to the degree of agreement with the statement. The cut-off point ≥ 33 was adopted for both sexes to classify a problematic smartphone user ^11^.

#### Sleep quality

Sleep quality was assessed by asking “How do you consider the quality of your sleep at the moment?”, with five possible answers (very good, good, regular, poor, and very poor), the categories “very good and good” and “regular, poor, and very poor” were grouped and transformed into the dichotomous variable good vs. poor sleep quality, respectively.

### Statistical analysis

Descriptive data analysis used absolute and relative frequencies for all the sociodemographic and behavioral variables. Pearson’s chi-square test was applied to compare the PSU between the variables of interest. The crude and adjusted Poisson regression with robust error verified the association of sociodemographic and behavioral factors with PSU. A second Poisson regression analysis was used to verify the association between PSU and sleep quality, adjusting for sex, skin color, and PAL. Interaction terms were created between PSU and gender, skin color, and PAL further to explore the associations between PSU and sleep quality. The prevalence ratio (PR) was used to measure association, and 95% confidence intervals (95%CI) were obtained for all the associations. Associations with a p-value < 0.05 were considered statistically significant for 2-tailed analyses. All analyses were conducted using Stata software, version 16.0 (https://www.stata.com).

## Results

The sample comprised 411 students (55.7% male) aged 15 to 22 (17.18 ± 1.50). Most participants reported having white skin (75.9%), attending physical education classes for 30 minutes or more (82.9%), and spending more time interacting with screens (46%). The minority of students reported being physically active (22.4%), while 37.2% reported a history of binge drinking (Table 1). There was no information missing from participants.

**Table 1:** Sociodemographic and behavioral characteristics of the high school students. Rio Grande, Rio Grande do Sul State, Brazil (n = 411).

Characteristics	n	%
Sex		
Male	229	55.7
Female	182	44.3
Age (years)		
15	73	17.8
16	64	15.6
17	98	23.8
18	94	22.9
19	59	14.4
≥ 20	23	5.5
Skin color		
White	312	75.9
Black	41	10.0
Yellow	3	0.7
Mixed-race	55	13.4
Socioeconomic status (quartiles)		
1 (lowest)	102	24.8
2	103	25.1
3	104	25.3
4 (highest)	102	24.8
PAL		
Inactive	128	31.1
Insufficiently active	191	46.5
Active	92	22.4
Participation in physical education class (minutes/class)		
< 30	70	17.1
≥ 30	341	82.9
Screen time (hours per day)		
Up to 2	63	15.3
> 2 to 4	159	38.7
> 4	189	46.0
Binge drinking		
No	258	62.8
Yes	153	37.2

PAL: physical activity level.

**Figure 1:**
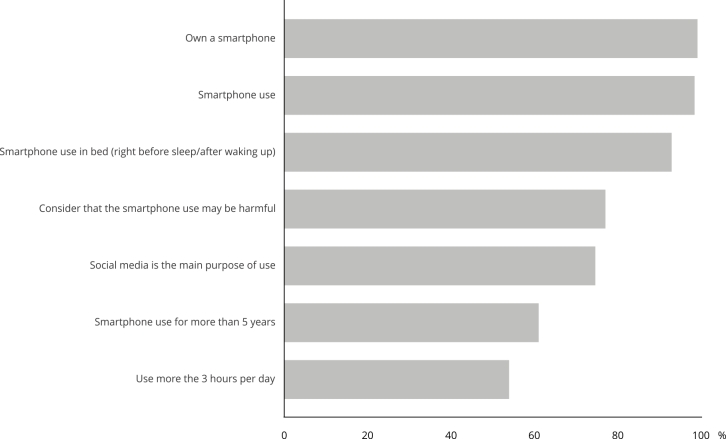
Characteristics and perception of smartphone use by high school students.

Figure 1 shows a descriptive analysis regarding the characteristics and perception of smartphone use. Almost every student uses a smartphone, whether it is their own device or not, with the following characteristics also standing out: have been using for more than five years (60.9%) despite their young age; browsing social networks as the primary purpose of use (74.5%); using it in bed before sleeping and/or as soon as they wake up (92.8%), and considering that use can be harmful (76.9%). PSU prevalence was 34.3% (95%CI: 29.6-38.9), and was higher among females (40.7%) than males (29.3%) (p = 0.02).

The crude analysis showed that female sex (PR = 1.38; 95%CI: 1.06-1.81), binge drinking (PR = 1.36; 95%CI: 1.04-1.77), and screen time (PR = 1.40; 95%CI: 1.14-1.70) were risk factors for PSU. The adjusted analysis kept the risk associations for PSU from the crude analysis but with different magnitudes, pointing at female sex (PR = 1.40; 95%CI: 1.06-1.85), binge drinking (PR = 1.35; 95%CI: 1.02-1.79), and screen time (PR = 1.48; 95%CI: 1.20-1.82). Higher socioeconomic status (PR = 0.88; 95%CI: 0.77-0.99) and longer participation in physical education classes (PR = 0.73; 95%CI: 0.54-0.99) showed a protective association for the PSU (Table 2).

**Table 2:** Prevalence of problematic smartphone use (PSU) and results of Poisson regression with crude and adjusted analysis for factors associated with PSU in high school students. Rio Grande, Rio Grande do Sul State, Brazil (n = 411).

Characteristics	PSU (%)	Crude analysis			Adjustedanalysis *		
		PR	95%CI	p-value	PR	95%CI	p-value
Sex							
Male	29.3	1.00			1.00		
Female	40.7	1.38	1.06-1.81	0.02	1.40	1.06-1.85	0.01
Age **	-	0.99	0.90-1.08	0.87	0.93	0.85-1.02	0.14
Skin color							
White/Yellow	35.2	1.00			1.00		
Mixed-race/Black	31.2	0.88	0.63-1.23	0.48	0.81	0.58-1.14	0.25
Socioeconomic status **		0.89	0.79-1.00	0.06	0.88	0.77-0.99	0.04
Bringe drinking							
No	30.2	1.00			1.00		
Yes	41.2	1.36	1.04-1.77	0.02	1.35	1.02-1.79	0.03
Screen time ***	-	1.40	1.14-1.70	< 0.01	1.48	1.20-1.82	< 0.01
PAL **	-	1.00	0.83-1.20	0.95	1.11	0.92-1.33	0.25
Participation in physical education class (minutes/class)							
< 30	42.8	1.00			1.00		
≥ 30	32.5	0.75	0.55-1.03	0.08	0.73	0.54-0.99	0.04

95%CI: 95% confidence interval; PAL: physical activity level; PR: prevalence ratio.

* Adjusted for gender, age, skin color, socioeconomic status, history of binge drinking, screen time, PAL, time spent in physical education classes;

** Age, socioeconomic status, PAL: ordinal variables. Age: in complete years; socioeconomic status: in quartiles (1 = lowest; 4 = highest); PAL: 1 = 0 minutes of physical activity/week; 2 = up to 299 minutes of physical activity/week; 3 = 300 minutes or more of physical activity/week;

*** Screen time: numeric variable (up to 2 hours per day [17.5% with PSU], ≥ 2 to 4 hours per day [33.3% with PSU], and > 4 hours per day [40.7% with PSU]).

Table 3 shows the association between PSU and sleep quality and the interaction between PSU and gender, skin color, and PAL. Students with PSU were more likely to report worse sleep quality than those without PSU (PR = 1.17, 95%CI: 1.02-1.34). Male students with PSU showed worse sleep quality (PR = 1.29; 95%CI: 1.07-1.59) than male students without PSU. Compared to white students without PSU, students with black and mixed-race skin color showed poorer sleep quality than students with white and yellow skin color (PR = 1.28; 95%CI: 1.06-1.54), which became even more prevalent when they had PSU (PR = 1.33; 95%CI: 1.05-1.69). Compared to physically active students without PSU, physically inactive students without PSU (PR = 1.30; 95%CI: 1.01-1.67) and with PSU (PR = 1.50; 95%CI: 1.13-1.98) had worse sleep quality. We further tested differences in the regression coefficients from the interaction analysis in Table 3, focusing on students with PSU (Table 4). The regression coefficients indicated that being physically active can moderate the impact of PSU on sleep quality in those who were classified as problematic smartphone users (p = 0.103)

**Table 3:** Association between problematic smartphone use (PSU) and sleep quality in high school students. Rio Grande, Rio Grande do Sul State, Brazil (n = 411).

Variables	Poor sleep quality		
	PR	95%CI	p-value
PSU *			
No	1.00		
Yes	1.17	1.02-1.34	0.02
PSU vs. Sex			
PSU = no and Sex = male	1.00		
PSU = no and Sex = female	1.12	0.93-1.34	0.22
PSU = yes and Sex = male	1.29	1.07-1.54	< 0.01
PSU = yes and Sex = female	1.18	0.96-1.44	0.11
PSU vs. Skin color			
PSU = no and Skin color = white/yellow	1.00		
PSU = no and Skin color = black/mixed-race	1.28	1.06-1.54	< 0.01
PSU = yes and Skin color = white/yellow	1.21	1.03-1.43	0.02
PSU = yes and Skin color = black/mixed-race	1.33	1.05-1.69	0.01
PSU vs. PAL			
PSU = no and PAL = active	1.00		
PSU = no and PAL = insufficiently active	1.05	0.81-1.37	0.68
PSU = no and PAL = inactive	1.30	1.01-1.67	0.04
PSU = yes and PAL = active	1.16	0.82-1.63	0.38
PSU = yes and PAL = insufficiently active	1.26	0.96-1.65	0.09
PSU = yes and PAL = inactive	1.50	1.13-1.98	< 0.01

95%CI: 95% confidence interval; PAL: physical activity level; PR: prevalence ratio.

* Adjusted for gender, age, skin color, socioeconomic status, excessive alcohol consumption, screen time, PAL, time spent participating in physical education classes.

**Table 4:** Interaction effect of problematic smartphone use (PSU) on sleep quality.

Interaction	p-value
PSU = Yes	
Sex: male vs. female	0.364
Skin color: white/yellow vs. black/mixed-race	0.371
PAL: active vs. insufficiently active	0.599
PAL: active vs. inactive	0.103

PAL: physical activity level.

## Discussion

This article verified the prevalence and factors associated with PSU in a sample of students from southern Brazil and explored how PSU was associated with sleep quality. We found that PSU occurred in more than one-third of the sample. Regarding sociodemographic variables, females were more likely to have PSU, while higher socioeconomic status students were less likely to have PSU. Concerning behaviors, binge drinking and longer screen time were considered risk factors for PSU, while longer participation in physical education classes was a protective factor. The students’ sleep quality was investigated as an outcome in a second analysis, and it was possible to verify associations with the PSU, with interactions permeated by gender, skin color, and PAL.

In line with previous studies, female adolescents seem to be excessively involved with their devices and are more prone to impulsive behavior ^21^^,^^22^^,^^23^. The relationship with the findings in international research is greatly influenced by access to specific content, such as social networking services, which are determining factors in generating problems with smartphone use among girls ^21^. However, there still needs to be more evidence linking females to the PSU in studies conducted in Brazil, shedding light on the possibility of filling this gap in the scientific field.

The protective association of higher socioeconomic status against PSU is not unanimously supported in the scientific literature, as both adolescents with higher and lower socioeconomic status can be prone to smartphone addiction ^24^. In federal institutes with similar characteristics to the IFRS throughout the country, 67.4% of students are in the upper half of the social class, which reflects their higher socioeconomic status ^25^. These individuals have greater opportunities for entertainment beyond the virtual possibilities offered by smartphone use and can fill their daily time with other activities (e.g., practicing individual and team sports, music lessons, trips, and outings). However, they might still make ill-adapted use of smartphones, suggesting that this behavior may contain extra elements that influence this relationship.

The investigation into the history of binge drinking and smartphone exposure in this study was in line with the results of a representative study with European adolescents ^26^. It is understood that excessive alcohol consumption among adolescents may be related to the search for social acceptance and affirmation towards maturity and the transition from childhood to adulthood ^27^. The harmful combination of both behaviors implies the urgency of pointing out the potential damage to health before adulthood, when alcohol consumption may occur more frequently because it is legally accepted, and the daily use of technology may be even more present among the individuals’ priorities.

High screen time was associated with PSU, although it did not address the isolated potential of smartphone exposure. The time spent using this device alone increases the chances of this use becoming problematic among young people, especially those who have been using smartphones since the age of 13 and spend more than 5 hours per day using the screen ^28^. Considering that high exposure must precede the possible repercussions of smartphone use, actions drawn up by guidelines such as the *Canadian 24-Hour Movement Guidelines*, which aim to reduce risk behaviors, are implemented and have positive results in the prevention of important health problems ^29^^,^^30^. In this sense, when looking at the Brazilian scenario, there is a lack of similar documents, suggesting that adopting similar strategies could be a valuable investment that already has theoretical and practical support.

Based on a general health perspective, physical education classes foster social interactions. These moments are capable of generating happiness, relieving stress, increasing physical and mental well-being, as well as promoting relationships with higher levels of physical activity among Brazilian adolescents ^31^^,^^32^. However, evidence suggests students prefer to use cell phones instead of practicing sports or social activities at school, which is a worrying scenario ^31^. Thus, the school can play a formative role by raising awareness about the possible harms of smartphone use. However, fundamentally, it must boost involvement in health promotion practices that benefit students beyond the school environment ^33^^,^^34^.

After observing the factors associated with PSU, we sought a better understanding of how it can be associated with important behavioral outcomes, in this case, sleep quality. Regarding biological aspects, we found that boys with PSU had worse sleep quality than boys without PSU. Some studies show particularities in boys’ relationship with the maladaptive use of smartphones, commonly using it for gaming ^35^^,^^36^. Interaction with this modality can impair their sleep quality, especially when used during nighttime ^37^. This reinforces the need for greater awareness of the adverse effects of using smartphones at night to achieve better sleep hygiene ^37^. Then, it is believed that it will be possible to prevent future problems regarding this critical behavioral aspect.

Concerning skin color, black and mixed-race students had worse sleep quality than white and yellow students without PSU, regardless of their PSU status. Controversially, this finding is specific to the investigated federal institute. In other words, it differs from a vital cohort study in southern Brazil, which results indicated that adolescents whose mothers had black skin color had a longer sleep duration when compared to white adolescents ^38^. This highlights the need to include other variables to better understand the state of sleep quality, which comprises a set of social aspects beyond adolescents’ skin color.

The scientific community should investigate aspects regarding the lifestyle of adolescents because the adoption of habits during childhood can continue into adulthood ^39^. Despite the recognized benefits of regular physical activity, physical inactivity is a worrying behavior, especially among youth, whose rate is nearly 80% ^40^^,^^41^. This study found that physically inactive adolescents have poorer sleep quality than active ones, and this association was intensified with adolescents classified as PSU. The regular practice of physical activity among adolescents establishes a starting point for probable positive health effects after its adherence, because physical activity prevents the development of maladaptive smartphone use during adolescence and improves sleep quality in later stages ^42^^,^^43^. This plausible behavioral network reinforces the importance of promoting physical activity for adolescents, both in and out of school, as it shows that physical activity can attenuate the impact of PSU on the sleep quality of this population.

Note that the convenience sample from this study does not enable the associations to be extrapolated to the entire population of high school students in Brazil. In addition, health researchers can use objective methods to assess sleep quality, such as actigraphy (considered the gold standard) ^44^, and subjective methods, such as the use of validated instruments ^45^. However, a single self-reported question was sued to assess sleep quality in this study. Regarding binge drinking, it must be revealed that questions about this behavior could bring memory and social desirability bias, influencing the quality and validity of the results.

Lastly, other variables of interest could be explored to provide even more robustness to the study, this perception being an important aspect to be considered in future studies. There are important findings showing that behavioral aspects, such as an unhealthy dietary pattern, consisting of snacks and ultra-processed foods ^46^, stricter and less close parent-child relationships seem to coincide with the longer use of smartphone and PSU during childhood and adolescence ^47^^,^^48^. Also, aspects of mental health such as depression, anxiety, and perceived stress may result in greater risk of PSU adoption ^10^.

Despite its limitations, this study makes a significant contribution to understanding the factors associated with PSU, and highlights the importance and the need for more research on this behavior in Brazil. In addition to the factors associated with PSU, the analysis of the interaction between PSU and sleep quality resulted in associations with subgroups of the sample, providing important information about the possible repercussions of PSU on aspects of the health of the investigated population. PSU is an emerging area for the scientific community, which may benefit from the theoretical contribution of this study in future research. Overall, this study provides support for disseminating more information about the repercussions and involvement of PSU in adolescents’ daily lives, a behavior that can be highlighted as a phenomenon with characteristics that can be modified, especially those related to lifestyle habits.

## Conclusions 

This study identified a significant rate of PSU among students, with around one in every three students having the behavior. In addition to the associations of PSU with sociodemographic and behavioral factors, adolescents with PSU have worse sleep quality, which can be potentially mitigated by adopting healthy habits, such as regular physical activity.
